# Stability and Bioaccessibility of Phenolic Compounds in Rosehip Extracts during In Vitro Digestion

**DOI:** 10.3390/antiox12051035

**Published:** 2023-04-30

**Authors:** Isabel Odriozola-Serrano, Danielle P. Nogueira, Irene Esparza, Ana A. Vaz, Nerea Jiménez-Moreno, Olga Martín-Belloso, Carmen Ancín-Azpilicueta

**Affiliations:** 1 Department of Food Technology, University of Lleida-Agrotecnio CERCA Center, Av. Alcalde Rovira Roure 191, 25198 Lleida, Spain; isabel.odriozola@udl.cat (I.O.-S.); olga.martin@udl.cat (O.M.-B.); 2 Department of Sciences, Institute for Advanced Materials (INAMAT2), Universidad Pública de Navarra, Campus Arrosadía s/n, 31006 Pamplona, Spain; danielle.pires@unavarra.es (D.P.N.); ancin@unavarra.es (C.A.-A.)

**Keywords:** rosehips, in vitro digestion, phenolic compounds, bioaccessibility, antioxidant activity

## Abstract

Rosehips, particularly dog rose fruits (*Rosa canina* L.), are a great source of antioxidant compounds, mainly phenolics. However, their health benefits directly depend on the bioaccessibility of these compounds affected by gastrointestinal digestion. Thus, the purpose of this research was to study the impact of gastrointestinal and colonic in vitro digestions on the concentration of total and individual bioaccessible phenolic compounds from a hydroalcoholic extract of rosehips (*Rosa canina*) and also their antioxidant capacity. A total of 34 phenolic compounds were detected in the extracts using UPLC-MS/MS. Ellagic acid, taxifolin, and catechin were the most abundant compounds in the free fraction, while gallic and *p*-coumaric acids were the main compounds in the bound phenolic fraction. Gastric digestion negatively affected the content of free phenolic compounds and the antioxidant activity measured using the DPPH radical method. However, there was an enhancement of antioxidant properties in terms of phenolic content and antioxidant activity (DPPH (2,2-diphenyl-1-picrylhydrazyl): 18.01 ± 4.22 mmol Trolox Equivalent (TE)/g; FRAP (Ferric Reducing Antioxidant Power): 7.84 ± 1.83 mmol TE/g) after the intestinal stage. The most bioaccessible phenolic compounds were flavonols (73.3%) and flavan-3-ols (71.4%). However, the bioaccessibility of phenolic acids was 3%, probably indicating that most of the phenolic acids were still bound to other components of the extract. Ellagic acid is an exception since it presented a high bioaccessibility (93%) as it was mainly found in the free fraction of the extract. Total phenolic content decreased after in vitro colonic digestion, probably due to chemical transformations of the phenolic compounds by gut microbiota. These results demonstrated that rosehip extracts have a great potential to be used as a functional ingredient.

## 1. Introduction

Consumers, nowadays, prefer a healthy and balanced diet based on natural products because it has been increasingly linked to overall health. Thus, the use of bioactive compounds to produce functional foods has been gaining momentum [1]. In this regard, rosehips have a potential interest for innovative food and medicinal applications thanks to their high concentration of bioactive compounds. This pseudo-fruit from the *Rosa* genus (*Rosaceae* family) comprises approximately 310 species spread throughout Europe, Africa, and Western and Northern Asia. It has been demonstrated that rosehips have strong antioxidant, antimicrobial, anti-inflammatory, antidiabetic, and anticarcinogenic activities [2,3]. Likewise, rosehips, particularly dog rose fruits (*Rosa canina* L.), have been traditionally used to prevent and treat rheumatoid arthritis [4]. This action has been validated by clinical studies with rosehip powder, which evidenced a reduction in the symptoms associated with rheumatoid inflammation [5]. In addition, Jiménez et al. [3] discovered that vitamin C and polyphenols from rosehips, when tested on cancer cells (Caco-2), presented antioxidant and antiproliferative effects. Therefore, rosehips are a promising source of functional ingredients to enrich food with nutritious and antioxidant substances, which is the reason for the growing interest in studying their bioactive composition and bioaccessibility. It is known that rosehips are rich in carotenoids, vitamins such as C, B1, B2, K, and E, amino acids, fatty acids, organic acids, and several polyphenols [6,7]. Rosehip seeds are also rich in fatty acids, the most abundant being polyunsaturated (73.88–79.52%), followed by monounsaturated (14.67–18.89%) and saturated fatty acids (5.22–7.36%) [8], although, their composition may be influenced by the *Rosa* species analyzed and by other factors such as the environment in which they grow (sun hours, amount of rain, altitude, and soil) [9].

Among the different natural bioactive substances contained in rosehips, phenolic compounds are of particular relevance as they can act as antioxidants, intervene in cell differentiation and carcinogen deactivation, and contribute to the maintenance and repair of DNA, among other important functions [10]. However, despite their well-known high potential for health, these compounds can interact with other molecules of the food matrix, such as proteins, digestible carbohydrates, lipids, and dietary fiber, compromising their bioaccessibility and, therefore, their bioavailability [11]. In this regard, there exist several studies on the phenolic composition of rosehips, but so far, none of them have analyzed the fraction of both free and matrix-bound phenolic compounds.

Bioaccessibility is defined as the fraction of a compound that is released from its matrix in the gastrointestinal tract, thus becoming available for intestinal absorption [12]. Therefore, the more bioaccessible a compound is, the greater its bioavailability, understood as the rate at which an active substance is absorbed and reaches the circulation. In fruit and vegetable matrixes, the bioavailability of antioxidants is determined by their low bioaccessibility due to physical and chemical interactions between antioxidants and the non-digestible polysaccharides of the cell walls [13]. In vitro methods that simulate the digestion process can be used to study the bioaccessibility of compounds from food sources and have some advantages compared to in vivo methods: they are faster, less expensive, safer, and without ethical restrictions [14]. In this way, Jara-Palacios et al. [15] studied the effect of in vitro gastrointestinal digestion on the phenolic composition and antioxidant activity of different white winemaking byproducts (grape pomace and its constituents: seeds, skins, and stems). In this work, the authors found that digestion reduced total polyphenolic content and antioxidant activity, although the Oxygen Radical Absorbance Capacity (ORAC) values of seed and stem extracts increased after the simulated digestion. Zhang et al. [16] evaluated the release and activity of polyphenols linked to soluble dietary fiber of wheat bran by in vitro gastrointestinal and colonic digestions. Their results suggest that the bioaccessibility of polyphenols linked to fiber was 7.42 times higher in colonic digestion than in the gastrointestinal phase. Gallic, *p*-hydroxybenzoic, and vanillic acids were the most abundant phenols after 6 h of colonic digestion. More recently, Vaz et al. [11] evaluated the bioaccessibility of phenolic compounds from some vegetable byproducts (artichoke, cucumber, red pepper, and carrot), finding a significant concentration of bioaccessible phenolic compounds after an in vitro gastrointestinal digestion. In addition, they also found a decrease in the total content of phenolic compounds after the in vitro colonic digestion, which evidenced the chemical transformation of these compounds by the gut microbiota. 

Thus, there were two main aims in the present work. On the one hand, to deepen the knowledge of the phenolic composition of rosehips in terms of free and matrix-bound phenolic substances. On the other hand, to evaluate the impact of gastrointestinal and colonic in vitro digestions on the bioaccessibility and antioxidant capacity of total and individual phenolic compounds from a hydroalcoholic extract from rosehips (*Rosa canina*). In this way, the potential of this extract to be used as a functional ingredient for food and nutraceutical applications will be evaluated.

## 2. Materials and Methods

### 2.1. Extracts

Rosehip fruits (*Rosa canina* L., *Rosaceae*) were provided by the company Herbes del Molí (Alicante, Spain), which has the necessary collection permissions. The fruits do not belong to an endangered species nor are they protected. The rosehips were collected in the mountain of Mariola in a landscape called “Ca’l retor”, located in the municipality of Agres (Alicante). Freshly collected rosehips were stored at −40 °C prior to analysis. Rosehips were dried in an air circulating oven at 30 °C until constant weight, and this was considered as dry weight. Afterwards, they were ground and sieved through a 300 µm mesh. Dry ground rosehips were stored in a plastic container in the fridge until use. The extracts were obtained by adding 200 mL of ethanol:water solution (1:1, *v*/*v*) to 2 g of powdered rosehips in Erlenmeyer flasks that were put in an oven at 40 °C for 24 h at 250 rpm (orbital shaker). Then, the solutions were centrifuged at 6000 rpm for 15 min and filtered through paper filters. The liquid was concentrated in a rotary evaporator at 40 °C. The concentrated extract was freeze-dried, and the resulting powder was stored under refrigeration in clear glass containers sealed with parafilm.

### 2.2. In Vitro Digestions

#### 2.2.1. Gastrointestinal Digestion

In vitro gastrointestinal digestion was carried out following the procedure described by Brodkorb et al. [17]. Samples from the two subsequent phases, gastric and intestinal, were considered. To simulate the gastric phase, rosehip extracts were mixed with simulated gastric fluids (SGF) containing pepsin (2000 U/mL in the final mixture), CaCl_2_ (0.3 M), Milli-Q water, and HCl (6 M). The mixture was placed in an incubator (Incubator OPAQ, OVAN, Barcelona, Spain) at 37 °C at 100 rpm for 2 h. After this time, 30 mL of this phase was transferred to a 100 mL beaker to simulate the intestinal phase, and 1.5 mL of intestinal salts solution (10 mM CaCl_2_ and 150 mM NaCl), 3.5 mL of bile solution (54 mg/mL), and 2.5 mL of pancreatin (75 mg/mL) were added. The pH of the mixture was adjusted to and maintained at 7 by adding NaOH (0.25 M) constantly for 2 h. Then, the total content was centrifuged at 14,000 rpm for 50 min at 4 °C. The supernatants were considered as the soluble portion of the simulated intestinal digestion (SPSID) and, therefore, the absorbable portion. The pellets were the insoluble portion of simulated intestinal digestion (IPSID). The supernatants were stored at −40 °C, and the pellets were lyophilized for 48 h for further colonic digestion.

#### 2.2.2. Colonic Digestion

A feces pool was obtained from 3 healthy adult donors, who had not taken antibiotics for at least three months prior to collection. This study was approved by the Ethics Committee of the Hospital Universitari Arnau de Vilanova, Lleida, Spain, CEIC-1980. To obtain the fecal slurry, the feces were diluted at a 1:10 (*w*/*v*) ratio in mineral medium (36.8 g/L FeSO_4_·7H_2_O, 19.9 g/L MnSO_4_·H_2_O, 4.4 g/L ZnSO_4_·7H_2_O, 1.2 g/L CoCl_2_·6H_2_O, 0.98 g/L CuSO_4_·6H_2_O, 0.17 g/L Mo_7_(NH_4_)_6_O_24_·H_2_O, 92.4 mg/L NaHCO_3_, 35.42 mg/L Na_2_HPO_4_·2H_2_O, 4.7 mg/L NaCl, 4.5 mg/L KCl, 2.27 mg/L Na_2_SO_4_·10H_2_O, 0.55 mg/L CaCl_2_, 1.0 mg/L MgCl_2_·6H_2_O, and 4.0 mg/L urea CO(NH_2_)_2_) with 15% glycerol, previously autoclaved and frozen at −80 °C before use [18]. 

For the colonic digestion, a 50 mL conic bottom sterile centrifuge tube was used, in which 4.5 mL of mineral medium (without glycerol, pH 7), 0.5 mL of fecal slurry, and 50 mg of the insoluble fractions after the intestinal digestion were added. Then, the tubes were put in an incubator with an orbital shaker at 100 rpm and 37 °C for 48 h in anaerobic conditions (BioMérieux^®^ S.A., Marcy-l’Étoile, France). After that, the tubes were stored at −40 °C.

### 2.3. Determination of Phenolic Compounds

The initial phenolic profile of rosehip extracts was determined following the method proposed by Mattila and Kumpulainen [19]. The free phenolic fraction was obtained from 0.5 g of freeze-dried extract that was added to a 50 mL centrifuge tube and sonicated for 30 min with 7 mL of solution (85 mL of 2 g/L of 2,(3)-tert-butyl-4-hydroxyanisole in methanol and 15 mL of 10% acetic acid in water (*v*/*v*)). Then, distilled water was added to reach 17 mL, and 2 mL of the mixture was filtered through a 0.22 μm nylon filter for the determination of free phenolic compounds. Bound phenolic compounds in rosehip extracts were determined after subsequent alkaline and acid hydrolysis. In this way, 12 mL of distilled water containing 1% ascorbic acid, 0.415% EDTA, and 10 mL of 10 M NaOH was added to the previous solution and stirred overnight at 20 °C for 16 h. After that, the pH was corrected to pH 2, and released phenolic compounds (esterified phenolic compounds) were extracted three times with 15 mL of cold diethyl ether and ethyl acetate (1:1) and centrifugation at 2000 rpm. The organic layers were combined, evaporated to dryness, dissolved into 2 mL methanol, and filtered through a 0.22 μm nylon filter. After the alkaline hydrolysis, 2.5 mL of concentrated HCl was added to the aqueous phase that was kept at 85 °C for 30 min. The diethyl ether and ethyl acetate extraction was similar to that for alkaline hydrolysis in order to determine conjugated phenolics (acid fraction).

To determine the free phenolic profile of gastric fraction, soluble and insoluble fractions of intestinal digestion, and colonic fraction, they were each mixed with 70% methanol in MilliQ water and homogenized with an Ultra-Turrax T25-Basic mixer at 1600 rpm for 120 s. Subsequently, the mixtures were sonicated with a Hielscher Ultrasonic Processor GmbH (mod UP4000S, Teltow, Germany) with a titanium tip H4 at 24 kHz and 125 μm nominal amplitude for 120 s to maximize phenolic extraction. Then, the contents were centrifuged at 12,500 rpm at 4 °C for 15 min, and the liquid portion was filtered through a 0.22 μm nylon filter. These phenolic extracts were stored at −40 °C in darkness until analysis. The identification and quantification of the individual phenolic compounds were performed in a UPLC-MS/MS on AcQuity Ultra-Performance TM liquid chromatography–tandem mass spectrometry equipment (Waters, Milford, MA, USA). The chromatographic conditions were as described by Delpino-Rius et al. [20]. Commercial standards were employed to quantify the individual phenolic compounds. Quantification was carried out using calibration curves for each analyzed compound (R^2^ ≥ 0.99). The samples were analyzed in triplicate, and the results were expressed as µg of phenolic compound/g of initial extract.

### 2.4. Determination of Antioxidant Activity

The antioxidant activity at each digestion stage was determined using DPPH and FRAP assays. The DPPH analysis was performed as described by Brand-Williams et al. [21]. 2,2-diphenyl-1-pycrilhydracyl (DPPH) was dissolved in methanol at a concentration of 0.09 mM, and the absorbance was adjusted to 0.9 ± 0.05 at 517 nm. The calibration standard solutions of Trolox had concentrations from 12.5 to 175.2 mg/L. A calibration curve was prepared for each specific digestion phase using the corresponding blank solutions in each case (at the same pH and with the same enzyme and other reagent additions). In all cases, the calibration curves had R^2^ ≥ 0.99. The samples were diluted with their corresponding blank, when necessary. For the DPPH antioxidant capacity determination, 150 µL of a sample was mixed with 2.85 mL of reagent, and the cuvette was kept in darkness for 30 min. The absorbance was read at 517 nm. The FRAP method was performed according the description of Benzie and Strain [22]. The FRAP solution consists of a mixture of 300 mM acetate buffer, 2,4,6-tris(2-pyridyl)-s-triazine 9.99 mM solution, and FeCl_3_·6H_2_O 20 mM solution, in the proportion 10:1:1. For the analysis, 2.85 mL of FRAP solution was homogenized with 150 µL of the sample. After 0.5 h in the dark, the absorbance was read at 595 nm. The calibration standard was also Trolox. The curve concentrations ranged from 12.5 mg/L to 250.3 mg/L, with three control standards inside the curve range of concentration. The R^2^ of the calibration curves was at least 0.99 in all cases. The samples and curves were also prepared using the specific blanks for each stage analyzed (at the same pH and with the same enzyme and other reagent additions). All the spectrophotometric analyses were performed in a UV-Vis spectrophotometer (Jenway 7315, Staffordshire, UK). In all cases, the samples were analyzed in triplicate, and the results were expressed as mmol of Trolox equivalent/g of initial extract.

### 2.5. Statistical Treatment

Digestions were conducted in duplicate, and three replicate analyses were carried out for each sample in order to obtain the mean value. The variance of the results was determined using the Kruskal–Wallis test. Pairwise comparisons using the Wilcoxon rank sum exact test were applied to ascertain the differences among means. The statistical treatment was performed using packages implemented in the R 4.2.2 software (R Core Team).

## 3. Results and Discussion

### 3.1. Characterization of Rosehip Extracts

#### 3.1.1. Antioxidant Capacity

**Figure 1:**
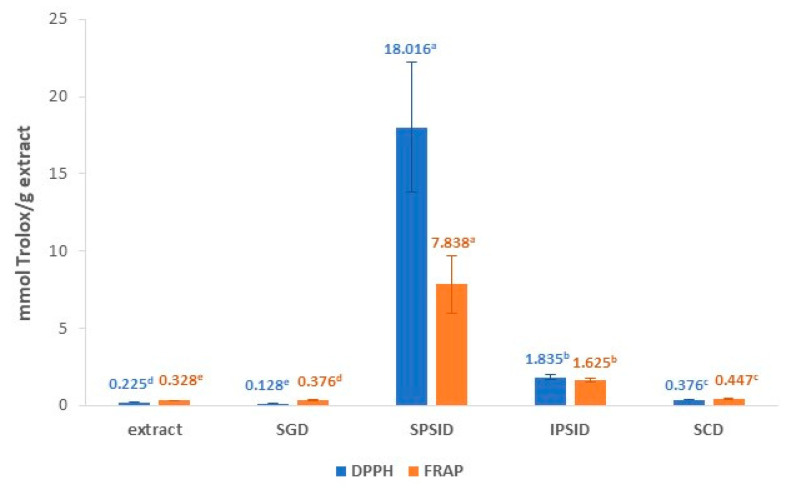
Antioxidant capacity of rosehip extract measured using the DPPH (2,2-diphenyl-1-picrylhydrazyl) and FRAP (Ferric Reducing Antioxidant Power) assays in different digestion stages. Values followed by different letters in the same color are significantly different at 95% confidence. (SGD = Simulated gastric digestion; SPSID = Soluble portion of intestinal digestion; IPSID = Insoluble Portion of Intestinal Digestion; SCD = Simulated Colonic Digestion).

The antioxidant capacity of the extracts was determined using DPPH and FRAP assays. These distinct methods were chosen in order to correctly evaluate the antioxidant activity of the extracts, as they have different action mechanisms, and the responses of the individual phenolic compounds differ among them [23]. As it can be seen in Figure 1, the antioxidant activity measured using the DPPH assay was 0.225 ± 0.026 mmol Trolox equivalent/g extract (0.328 ± 0.01 mmol Trolox equivalent/g rosehips), while measured using the FRAP assay it was 0.328 ± 0.010 mmol Trolox equivalent/g extract (0.255 ± 0.026 mmol Trolox equivalent/g rosehips). There are few studies about the antioxidant capacity of ethanolic extracts from rosehip, and those that exist use different methods than those described in this work, as there is no standardized method in the literature to characterize the antioxidant potential of an extract. However, comparisons can be made with extracts obtained and analyzed using other methods. For instance, Su et al. [24] analyzed the antioxidant activity by ABTS (2,2′-azino-bis(3-ethylbenzothiazoline-6-sulfonic acid) and ORAC (Oxygen Radical Absorbance Capacity) of 50% acetone and 80% methanol extracts of rosehips, among other botanicals. They found ABTS values of 0.379 ± 0.003 mmol Trolox equivalent/g rosehips (50% acetone) and 0.190 ± 0.005 mmol Trolox equivalent/g rosehips (80% methanol), which were of the same order as the values obtained in the present study. However, the ORAC results were higher, 0.838 ± 0.074 mmol Trolox equivalent/g rosehips (50% acetone) and 1.085 ± 0.024 mmol Trolox equivalent/g rosehips (80% methanol). The difference can be attributed not only to the different method but also to the extraction procedure and the intrinsic characteristics of the botanical material [7,24].

#### 3.1.2. Individual Phenols

**Figure 2:**
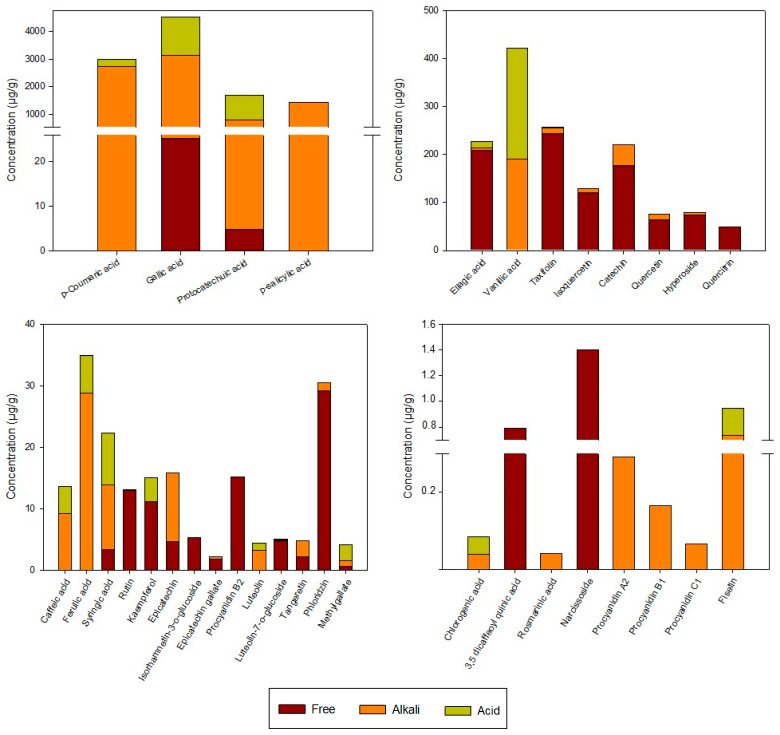
Initial phenolic profile (µg/g) of rosehip extracts in free, alkali, and acid fractions.

The individual phenolic compounds in the rosehip extracts analyzed using UPLC are shown in Figure 2. Total phenolic compounds (TPC) in the extracts, which were calculated as the result of the sum of free and bound phenolic compound, were 12,370 µg/g. Goztepe et al. [25] reported TPC values, also determined using HPLC, in different dry rosehips from Turkey (9056–10,523 µg/g), similar to those obtained in this study. Four different groups of phenolic compounds were detected in the extracts, and those with the highest content were phenolic acids (11,375 µg/g), followed by flavonols (628 µg/g) and flavan-3-ols (314 µg/g), while the lowest content was that of flavone compounds (18 µg/g). Thus, about 92% of the phenolic compounds present in the extracts are phenolic acids. In agreement, Demir et al. [26] found that phenolic acids are the most abundant phenolic compounds in *Rosa canina* fruits, followed by flavonoids. However, most of the authors analyzing phenolic compounds in rosehip fruits reported that the main compounds were flavonoids, probably because the covalently bound forms were not quantified in these research works [25,27]. The phenolic compounds in the extracts were determined in three fractions: the free phenolic compounds, the esterified fraction determined after an alkaline hydrolysis, and the conjugated fraction determined after acid hydrolysis. In this sense, it should be noted that the phenolic compounds considered to be present in their free form were those that were detected in the extract without subjecting it to any type of hydrolysis. Most of the phenolic compounds found in the extracts were in the esterified (8407.7 µg/g) rather than free (1130.2 µg/g) or conjugated (2832.1 µg/g) form. This is consistent with previous studies that demonstrated that alkaline hydrolysis is more efficient in releasing the bound phenolic compounds than acid hydrolysis [28]. The main compounds in the esterified and conjugated fractions were phenolic acids, representing 98.8% of total alkaline and 99.7% of the acid fractions. Flavonols and flavan-3-ols were mainly detected in free forms with values of 589 µg/g and 259 µg/g, respectively. It has been suggested that most of the phenolic acids In rosehips from *Rosa rugosa* are esterified or glycosidically linked, whereas flavonoids are usually found in free form [29]. However, information about the content of free and bound phenolic compounds in *Rosa canina* L. fruits is still not available. 

Regarding phenolic acids, gallic acid was the main phenolic acid in the alkali and acid fractions of the extracts, suggesting that this compound could be found mainly linked to cell matrix components by ester (released by alkaline hydrolysis) or glycosidic bonds (released by acid hydrolysis). In this way, Jiménez et al. [30] reported that gallic acid is liberated as a result of the alkaline and acid hydrolyses from gallotannins and gallolyl esters in rosehip fruits. Huang et al. [31] also found gallic acid as the main phenolic acid in *Rosa roxburghii* extracts, mainly bound to the matrix. However, in the case of ellagic acid, quercetin, and catechin, they found higher contents in their bound form than in their free form, which is not in agreement with the results obtained in the present study. These differences could be related to the plant species used as raw material and the extraction procedure performed. 

Other phenolic acids detected at a high concentration as ester-bound or glucoside forms were *p*-coumaric, *p*-salicylic, protocatechuic, and vanillic acids, whereas ellagic acid was the main phenolic acid in the free fraction (Figure 2). These results agree with those reported by Goztepe et al. [25], who found that gallic, ellagic, vanillic, and protocatechuic acids were the most representative phenolic acids in rosehips. In addition, Ghendov-Mosanu et al. [32] reported that *p*-salicylic acid is one of the most abundant phenolic acids in rosehip dry powder. On the other hand, other minor phenolic acids, such as caffeic, chlorogenic, 3,5-dicaffeoylquinic, ferulic, rosmarinic, and synringic acids, were also detected in the extracts but accounted for less than 1% of the total phenolic acids. 

Quercetin and its derivatives were the main flavonols identified in the extracts as free, ester-bound, or glucoside-bound forms, representing just 5% of total phenolic compounds. Among them, taxifolin (also known as dihydroquercetin) was the most abundant (257.5 µg/g). Substantial amounts of isoquercetin (128.6 µg/g), hyperoside (78.6 µg/g), quercetin (76.4 µg/g), quercitrin (53.7 µg/g), rutin (15.7 µg/g), and kaempferol (11.1 µg/g) were also detected. However, only relatively low amounts of narcissoside and isorhamnetin-3-O-glucoside were found in the extracts. Consistently, several authors also detected quercetin, rutin, isoquercetin, quercitrin, and kaempferol in *Rosa canina* species [25,27,33]. The extracts analyzed in this work did not contain myricetin, despite the fact that other studies have detected its presence in rosehips [29,34]. To our best knowledge, few authors detected taxifolin, narcissoside, or hyperoside in dog rose fruits [35,36]. Differences in the content of phenolic compounds could be related to environmental factors, such as light, temperature, and soil nutrients, as well as the fruit ripeness, which may affect the metabolism and conversions of phenolic compounds [34]. 

As described in the cited literature, flavan-3-ols are another relevant phenolic compound in rosehips, usually accumulated as catechin and B-type procyanidins [26]. In this study, the flavan-3-ols profile revealed that catechin was the main compound (219.6 µg/g), representing 70% of the compounds identified in this phenolic group. The other flavan-3-ols detected in substantial amounts in their free form were procyanidin B1 (56.7 µg/g) and B2 (15.2 µg/g), whereas epicatechin was found at 70.5% in an ester-bound form and 29.5% in a free form. Epicatechin gallate and procyanidin A2 were found in very small amounts in alkaline fractions, with values of 1.96 µg/g and 1.49 µg/g, respectively. 

Considering the flavones, luteolin and its glycoside forms were the main compounds in the three analyzed fractions of the undigested extracts (Figure 2), suggesting that they could be found both free and linked to cell matrix components by ester or glycosidic bonds. In addition, the polymethoxylated flavones tangeretin and fisetin were also detected in the extracts, representing 39% of the flavones. In contrast, Elmastaς et al. [27] detected apigenin-7-glucoside and naringenin as the main flavones in *Rosa canina* species at different harvesting times. The dihydrochalcone phlorizin, which has been found in different rosehip species [37], has also been detected in the extracts at a concentration of 30.5 µg/g. 

### 3.2. Effect of Gastrointestinal and Colonic Digestion on Total and Individual Phenolic Content and Antioxidant Properties of Rosehip Extracts

Concerning antioxidant activity, when extracts were measured using the FRAP method, a significant increase of 14.6% was observed from undigested samples to those subjected to gastric digestion (Figure 1). However, besides the increase in FRAP values, a significant decrease in DPPH values (43.1%) was found after the simulated gastric digestion (SGD) of the extracts. It is important to take into consideration that the FRAP assay is conducted in a buffered medium. This means that the variations in antioxidant capacity found in this medium are exclusively due to the change in the concentration of antioxidant compounds and not to the influence that the pH exerts on their structure and, therefore, on their antioxidant capacity [38]. However, the DPPH method was not conducted in buffered medium, and it has been shown that, among other factors, pH can affect the rate and pathways of the DPPH reaction with antioxidants [39]. This inconsistent behavior pattern between both methods could be explained by considering that alterations in the structure of antioxidants after digestion may affect their reactivity in the DPPH assay [40], being electron transfer agents significantly more affected by pH modifications than hydrogen atom donors [39]. In addition, this method has another limiting factor related to the steric accessibility of some phenolic compounds [39]. Thus, it can be easily reported that, depending on the antioxidant compounds released and/or degraded after gastrointestinal digestion, the DPPH values can either be correlated or not correlated with the FRAP values. 

The soluble portion of intestinal digestion (SPSID) had the highest antioxidant activity when compared to the other digestion stages, with DPPH and FRAP values of 18.0 ± 4.2 and 7.8 ± 1.8 mmol Trolox equivalent/g extract, respectively. This increase agrees with previous works, which explained that the change from acidic to alkaline pH from gastric to intestinal simulated digestions may increase the antioxidant activity by the deprotonation of the hydroxyl moieties present in the aromatic rings of the phenolic compounds [41,42]. On the other hand, Correa et al. [43] tested in vitro digestion of yerba mate extracts and suggested that there may be variations in the concentrations of phenols caused by chemical reactions among the compounds, which are highly sensitive to alkaline conditions. Most likely, these reactions vary in different batches, and this may explain the high standard deviation observed at this stage. This hypothesis was supported by Sirisena et al. [44], who stated that phenols are a large group of compounds known to suffer irreversible structural changes at alkaline pH by isomerization, auto-oxidation, and conjugation, with some being more susceptible than others, depending on their structures. At the same time, an enhancement of total free phenolics was observed in this fraction in comparison to the SGD, reaching a value similar to that of the undigested extracts. Chait et al. [45] also observed an increase in total free phenolic compounds after the intestinal phase digestion of carob powder, which they linked to the effect of digestive enzymes and bile salts interacting with the food matrix to release bound phenolic compounds. This agrees with the increase in the content of free phenolic compounds previously observed after the alkaline hydrolysis of the undigested extracts, which confirms that this type of hydrolysis is more efficient in releasing the bound phenolic compounds than acid hydrolysis [28]. However, this increase in total free phenolics is very moderate in comparison with the high increase in antioxidant capacity values found after digestion in intestinal conditions. The lack of correlation between the total content of phenolic compounds and antioxidant capacity may be due to the presence of high levels of other non-phenolic compounds in rosehips with antioxidant capacity, such as ascorbic acid, vitamin E, and carotenoids [9,13,46]. 

The intestinal bioaccessible index (BI) of total phenolic compounds present in the extracts (calculated as a percentage of the phenolic concentration in the SPSID of the total concentration in the undigested extract) was just 9% (Table 1). This fact suggested that the majority of phenolic compounds found in ester and glycoside forms in the undigested fraction are still bound to different components of the extracts or have been degraded during intestinal digestion. It has been reported that phenolic compounds can be degraded, precipitated, and become unstable during the intestinal phase as a result of the effects of enzymatic action and pH on hydroxyl groups [47]. These changes are mostly related to isomerization, hydrolyzation, protein binding reactions, and complexation with pancreatin proteins, forming high molecular weight phenolic derivatives with low solubility [48]. Several authors have reported higher intestinal bioaccessibilities of phenolic compounds (from 25% to more than 100%) in different fruit extracts than those found in this study [11,47]. However, in these works, intestinal bioaccessibility was calculated by considering just the free phenolic content of the fruits and underestimating the TPC as the sum of free and bound phenolic compounds.

On the other hand, the insoluble portion of simulated gastrointestinal digestion was used for simulated colonic digestion in order to evaluate if the phenols that still remain in the extract after digestion have the potential to be released in the colon (results presented in Table 1). The insoluble portion of the simulated intestinal digestion (IPSID) still retained antioxidant activity, but the DPPH and FRAP values significantly dropped from the IPSID to colonic stage (Figure 1). Low amounts of free phenolic compounds (17.6 µg/g) were detected using UPLC in the IPSID, indicating that most of the phenolic compounds in rosehip extracts do not reach the colon or were still bound to the food matrix (Table 1). In addition, the colonic digestion step drastically decreased the concentration of individual phenolic compounds (≈83%); thus, the depletion in antioxidant activity was related to this decrease. This fact agrees with previous studies that found a relevant degradation of different phenolic compounds after 24 h of fecal fermentation [44]. Accordingly, Zhang et al. [16] confirmed that most of the bound phenolic compounds cannot be hydrolyzed by digestive enzymes but can be released and metabolized by the enzymes of intestinal bacteria. The low antioxidant activity found using the DPPH and FRAP assays could indicate that the fecal slurry bacteria transformed the phenols in other phenolic compounds with lower antioxidant capacity, as polyphenols are known to have higher antioxidant activities than monophenols [11,21,43]. 

The effect of gastrointestinal digestion was dependent on the chemical class of the phenolic compounds. Regarding phenolic acids, the total free content decreased significantly (36%) during SGD but was enhanced after the intestinal phase, reaching a free phenolic acid content higher than that determined in the undigested extracts. However, the BI of phenolic acids was around 3% (Table 1). Phenolic acids in free form are very rarely present in plants, and the majority of phenolic acids, which are covalently bound to the matrix, are difficult to release during gastrointestinal digestion [48]. In the same way, Zhang et al. [16] and Pérez-Jiménez et al. [49] indicated that the phenolic compounds closely bound to the cell matrix, such as phenolic acids, are less affected by the neutral environmental pH and the enzymes acting in the small intestine. 

The total free content of flavan-3-ols and flavones dropped after SGD, followed by an increase after the intestinal phase, but did not reach the initial content at any time. Gastrointestinal digestion had a significant effect on free flavonol content, gradually decreasing the content of free phenolic compounds from 14% after SGD to 22% in the SPSID. The most bioaccessible classes of phenolic compounds were flavonols and flavan-3-ols, with BI values of 73% and 72%, respectively. Previous studies indicated that in vitro digestion processes led to a drastic qualitative and quantitative reduction in phenolic compounds, also affecting their bioaccessibility [15,43,50]. In accordance with our results, flavonoids presented higher bioaccessibility than phenolic acids in washed carob flours [51] and grapes [38]. 

Not only did the total phenolic acid concentration vary during gastrointestinal digestion compared to the undigested extracts, but the profile of these compounds was also affected. Some phenolic acids found in small amounts in undigested rosehip extracts, such as 3,5 dicaffeoylquinic, ferulic, and rosmarinic acids, were not detected in free form after gastrointestinal digestion. Thus, these phenolic compounds might be degraded due to the gastrointestinal fluids. In addition, gastric and intestinal phases had a strong effect on some minor phenolic acids since their content increased significantly after these stages.

Phenolic acids such as caffeic, chlorogenic, and *p*-coumaric acid, which were not detected in the free fraction of undigested extracts, were found in the SGD (0.83–2.55 µg/g) and the SPSID (1.8–65.3 µg/g). In addition, the high BI (148%) obtained for chlorogenic acid should be noted. A plausible explanation for this high value could be related to the fact that gastrointestinal conditions might facilitate the degradation of 3,5 dicaffeoylquinic acid, producing chlorogenic acid (3-caffeoylquinic acid) as a product [52]. Ellagic acid, which was found mainly in free form in the undigested extract, diminished significantly during SGD, but its content increased in SPSID (BI of 93.4%). Similar behavior was observed in gallic acid, although a low BI was obtained after the gastrointestinal tract for this compound (BI: 0.68%). Thus, physiological conditions such as those used in this study are not able to release gallic acid as a major hydrolytic product from gallotannins. Similarly, the low BI values of protocatechuic acid (2.5%), *p*-coumaric acid (2.2%), and syringic acid (7.6%), as well as the fact that vanillic acid was not detected neither after SGD nor in SPSID, indicate that these phenolic acids could still be bound to the extract matrix by ester or glycoside bounds. On the other hand, *p*-salicylic acid showed a very different trend from most of the phenolic acids detected in the extracts. Although the free *p*-salicylic acid content determined in SGD (1.4 µg/g) was higher than that analyzed in the undigested extracts, this acid was not detected in SPID, which is in accordance with a previous study that reported that salicylic acid in an exotic fruit was degraded under intestinal conditions [53]. 

The content of the most abundant free flavonol in the extracts, taxifolin, was significantly reduced during gastrointestinal digestion, reaching a BI of 61.1% in the intestinal phase. A higher reduction and, in turn, lower BI values were obtained for the free aglycone quercetin (43.2%). Bermúdez et al. [54] reported a complete degradation of this flavonol in chokeberry after an in vitro duodenal stage. It has been suggested that the instability of quercetin during digestion is related to its hydroxyl substitutions [55]. By contrast, some flavonol glycosides, such as isoquercetin and quercitrin, remained stable during the SGD and intestinal phase, with a BI of 86.8% and 107%, respectively. This could be due to the fact that sugar substitution in flavonoid molecules increases their stability during gastrointestinal digestion due to the interaction between the glycoside forms and sugar residues in soluble fiber and/or carbohydrates present in the extracts [56]. In agreement with our results, several authors have suggested that the in vitro bioaccessibilities of the flavonoids’ glucoside forms are higher than those obtained for the aglycone forms [50,56].

In the case of flavan-3-ols, the effect of gastrointestinal digestion varied depending on the structure and the polymerization degree. The concentrations of the free monomeric flavonols and procyanidins were significantly reduced after SGD compared to those in the undigested extracts, with the exception of epicatechin gallate and procyanidin A2, whose concentrations were increased, and procyanidin C, which remained stable under acidic conditions. It has been reported that gastric digestion can release monomeric flavan-3-ols from dimeric or trimeric compounds due to the acidic pH reached in the stomach [57]; however, this trend was not observed in our study. In addition, some studies have shown that the stability of procyanidins is related to the environmental pH, and it is higher in acidic conditions than under neutral or alkaline conditions. In fact, catechin significantly dropped from the stomach to the intestinal phase, which is consistent with the findings of Neilson et al. [58]. Small intestine conditions are particularly favorable for catechin degradative reactions. High pH, residual dissolved oxygen, and the likely presence of reactive oxygen species from normal digestive function may facilitate several reactions, including epimerization and auto-oxidation in the intestinal lumen [59]. Interestingly, procyanidin C1 was not detected after intestinal digestion, exhibiting lower stability than dimeric procyanidins. Yan et al. [60] suggested that procyanidin C1 is less stable than dimeric procyanidins because of its additional C4-O-C8 bond. At the same time, the dimeric procyanidin B1 content rose from the stomach to the intestinal stage, reaching BI values of 213%. Li et al. [61] concluded that increasing pH levels above 4 could enhance the production of *trans*-configured catechins, suggesting that isomerization was more favored in basic conditions. Thus, intestinal conditions might facilitate not only the release of dimeric procyanidins B1 from trimeric C1 but also the conversion of the *trans*-configured forms into the *cis*-isomers. 

Considering flavones, luteolin as an aglycone and the polymethoxylated flavone fisetin were not detected neither after SGD nor in the SPSID, whereas luteonin-7-O-glucoside remained stable in acidic conditions. A particular interest should be paid to tangeretin behavior, since after suffering a significant reduction in gastric phase (not detected), its concentration rose up to 1.9 µg/g (BI = 39.4%). This increase in the intestinal stage might indicate that intestinal conditions favor its release from rosehip extracts. Nevertheless, the BI of flavones (50%) was lower than those found in other flavonoids groups, which agrees with the results observed in cocoa powder by Ortega et al. [62]. Considering other phenolic compounds, it should be noted that dihydrochalcone phlorizin was found in the SGD and SPSID samples at a significantly higher concentration than that detected in the undigested extracts. This could mean that not all of the matrix-bound phlorizin was released after acid and/or basic hydrolysis in undigested extracts or that it underwent degradation under these conditions. In addition, methyl gallate, which was mainly matrix-bound in the undigested samples, presented a BI of 97.6%. Similarly, Ordóñez-Díaz et al. [63] found a significant increase in the content of methyl gallate in mango pulp as a consequence of the breakage of the weak bond between this compound and the food matrix by the action of pancreatin and bile salts. 

The main phenolic compounds detected in the IPSID were flavonols and flavan-3-ols, accounting for 78.7% of the total concentration. Significant reductions in the content of both groups (≥91%) were observed after simulated colonic digestion (SCD) compared to those analyzed in the IPSID, meaning that these compounds have been transformed into other phenolic metabolites by the microorganisms present in the gut. Polymeric procyanidins B2 and C1 and the glycoside forms hyperoside, rutin, and isorhamnetin-3-O-glucoside, which were identified in the IPSID, were not detected after SCD. Gut microbiota enzymes such as glucosidases and rhamnosidases are involved in the deglycosylation of flavonoids, releasing the sugar moiety and generating aglycones, which can be further catabolized to low molecular weight compounds. On the other hand, Urpí-Sardà et al. [64] reported that the polymeric procyanidins (mainly B and C) which reach the colon are transformed into hydroxyphenyl propionic acids and hydroxyphenyl valerolactones by the gut microbiota. However, A procyanidins, which contain an additional ether bond between the C2 of the upper unit and the oxygen-bearing C7 of the lower unit, are more stable in the colon since microbiota have a limited capacity to degrade these polymeric compounds [65]. 

By contrast, the total content of phenolic acids and flavones was more or less stable after simulated colonic digestion. Some phenolic acids, such as caffeic, protocatechuic, syringic, and *o*-salicylic acid, exceeded the concentration found in the IPSID after SCD, suggesting that these phenolic acids could be generated by colonic bacteria from other phenols or have been released from the extracts. Interestingly, vanillic acid was not found in the extracts in the colonic stage, which could be related to the fact that vanillic acid is converted to protocatechuic acid by gut microbiota with the help of vanillate O-demethylase [66]. The bacterial enzymes deglycosylate different compounds, but the microbiota can also perform a range of other transformations, including oxidation, demethylation, and catabolic degradation to smaller fragments, including small phenolic acids and aromatic compounds [67]. 

In summary, this in vitro digestion model has allowed us to estimate, for the first time, the bioaccessibility index of the phenolic compounds from rosehip extracts and to evaluate the influence of the different digestion conditions on each of them. This study evidenced that there are several phenolic compounds that can potentially be absorbed and used by the organism after the digestion of these extracts. However, it must be taken into consideration that not all soluble compounds are absorbed by the organism [68], as they can be influenced by several environmental factors that occur in vivo (mechanical forces that contribute to in vivo digestion and nutrient release, hormonal and neural control, biochemical changes, metabolite accumulation that can interfere with digestion, transport dynamics, interferences from other food components, and mucosal cell activity), which cannot be assessed in static models of in vitro digestion [68,69]. Therefore, the results reported in the present study are very useful for predictive purposes and should be completed with future in vivo assays. 

## 4. Conclusions

The concentration of phenolic compounds in free, conjugated, and esterified forms of rosehip ethanolic extracts from *Rosa canina* fruits, as well as the effect caused by a simulated in vitro gastrointestinal and colonic digestion, were investigated for the first time. The phenolic compounds in the extracts were mainly esterified phenolic acids, accounting for 67% of the total phenolic compounds. Both the phenolic content and the antioxidant activity of rosehip extracts significantly changed in the different digestion stages. These changes may result from the different stability levels of phenolic compounds and the release of matrix-bound compounds during simulated in vitro digestion. Among all the phenolic compounds studied, flavonols and flavan-3-ols demonstrated to be highly bioaccessible and, therefore, they could exert their biological activity in the organism. However, most of the phenolic acids, despite being present in high amounts in plant extracts, are poorly bioaccessible and, thus, their biological effects will be more limited. Ellagic acid was an exception, as it was present in considerable amounts mostly in the free fraction of the extracts and, therefore, showed a high BI (93%). Chlorogenic acid also presented a high BI, but the total amount of this compound was very low. Thus, rosehip extracts can be considered a good source of highly bioaccessible flavonols (especially taxifolin), flavan-3-ols (especially procyanidin B1), and ellagic acid. On the other hand, it is important to point out that the glucoside forms of flavonoids showed higher bioaccessibility than their corresponding aglycones, probably due to their high stability during the digestion process. For this reason, although flavonoids’ glucosides have lower antioxidant capacity than aglycones, their greater stability and bioaccessibility make them more interesting from a biological point of view. Nevertheless, further studies are needed to investigate the bioaccessibility of other antioxidant compounds of rosehips, such as ascorbic acid, carotenoids, and vitamin E, to complete the identification of the main components of rosehip extracts responsible for their health benefits. In addition, in vivo studies (with animal and human models) are required to know the bioavailability of phenolic compounds from rosehip extracts and predict their potential biological effects. 

## Figures and Tables

**Table 1:** Effect of gastrointestinal and colonic digestion on free phenolic profile of rosehip extracts.

	Initial (Free Fraction) (µg/g)	SGD ^1^ (µg/g)	SPSID ^2^ (µg/g)	BI ^3^ (%)	IPSID ^4^ (µg/g)	SCD ^5^ (µg/g)
ACIDS						
Caffeic acid	Nd	0.83 ^b^	1.8 ^a^	13.4	0.016 ^d^	0.060 ^c^
Chlorogenic acid	Nd	2.2 ^a^	2.7 ^a^	148	0.013 ^b^	0.072 ^b^
p-Coumaric acid	Nd	2.5 ^b^	65.3 ^a^	2.2	0.118 ^c^	0.075 ^c^
3,5-Dicaffeoylquinic acid	0.79	nd	nd	-	nd	nd
Ellagic acid	208.0 ^a^	100.7 ^b^	212.3 ^a^	93.4	2.115 ^c^	0.936 ^d^
Ferulic acid	Nd	nd	nd	-	nd	nd
Gallic acid	25.2 ^a^	13.9 ^b^	30.9 ^a^	0.68	0.247 ^c^	0.085 ^d^
Protocatechuic acid	4.9 ^b^	32.1 ^a^	43.3 ^a^	2.5	0.163 ^d^	0.397 ^c^
Rosmarinic acid	Nd	nd	nd	-	nd	nd
p-Salicylic acid	Nd	1.4 ^a^	nd	-	0.111 ^b^	0.042 ^c^
o-Salicylic acid	Nd	nd	nd	-	nd	0.235
Syringic acid	3.4 ^a^	1.4 ^c^	1.7 ^b^	7.6	0.048 ^e^	0.122 ^d^
Vanillic acid	Nd	nd	nd	-	0.103	nd
Total phenolic acids	242	155	358	3	2.934	2.024
FLAVONOLS						
Quercetin	64.6 ^a^	43.0 ^b^	33.0 ^c^	43.2	1.285 ^d^	0.114 ^e^
Taxifolin	243.7 ^a^	189.9 ^b^	155.6 ^b^	61.1	2.101 ^c^	0.089 ^d^
Isoquercetin	120.2 ^a^	109.0 ^a^	111.7 ^a^	86.8	2.129 ^b^	0.059 ^c^
Hyperoside	73.6 ^b^	83.0 ^a^	74.8 ^b^	95.2	2.446 ^c^	nd
Quercitrin	53.7 ^a^	56.3 ^a^	57.4 ^a^	107	0.687 ^b^	0.037 ^c^
Kaempferol	11.1	nd	nd	-	nd	nd
Rutin	15.6 ^b^	19.3 ^a^	21.8 ^a^	138	0.179 ^c^	nd
Narcissoside	1.4 ^b^	1.8 ^a^	1.7 ^ab^	121	0.020 ^d^	0.032 ^c^
Isorhamnetin-3-O-glucoside	5.4 ^a^	4.7 ^a^	4.5 ^a^	83.3	0.163 ^b^	nd
Total flavonols	589	507	460	73	9.010	0.331
FLAVAN-3-OLS						
Catechin	177 ^a^	109.7 ^b^	91.8 ^c^	41.9	2.857 ^d^	0.140 ^e^
Epicatechin	4.7 ^a^	1.8 ^c^	2.4 ^b^	15.2	0.070 ^d^	0.051 ^d^
Epicatechin gallate	1.5 ^b^	1.9 ^a^	1.8 ^a^	92.3	0.081 ^c^	nd
Procyanidin A2	1.1 ^b^	1.5 ^a^	1.5 ^a^	104	0.173 ^c^	0.165 ^c^
Procyanidin B1	56.5 ^b^	42.1 ^b^	120.7 ^a^	213	1.420 ^c^	0.063 ^d^
Procyanidin B2	15.2 ^a^	9.3 ^b^	6.1 ^b^	40.1	0.155 ^c^	nd
Procyanidin C1	2.6 ^a^	3.7 ^a^	nd	-	0.066 ^b^	nd
Total flavanols	259	170	224	72	4.822	0.419
FLAVONES						
Luteolin	Nd	nd	nd	-	nd	Nd
Luteolin-7-O-glucoside	7.8 ^a^	7.4 ^a^	7.5 ^a^	92.6	0.121 ^b^	Nd
Tangeretin	2.3 ^a^	nd	1.9 ^b^	39.4	nd	0.114 ^c^
Fisetin	Nd	nd	nd	-	nd	Nd
Total flavones	10	7	9	50	0.121	0.114
OTHERS						
Phlorizin	29.2 ^b^	52.6 ^a^	46.7 ^a^	153.1	0.531 ^c^	Nd
Methyl gallate	0.70 ^c^	1.1 ^b^	4.1 ^a^	97.6	0.153 ^d^	0.074 ^e^
TOTAL FREE PHENOLIC COMPOUNDS	1130	893	1103	9	17.571	2.962

^1 ^SGD = Simulated Gastric Digestion; ^2^ SPSID = Soluble Portion of Intestinal Digestion; ^3^ BI = Bioaccessibility Index; ^4^ IPSID = Insoluble Portion of Intestinal Digestion; ^5^ SCD = Simulated Colonic Digestion; different letters in the same row indicate that the mean values are significantly different (*p* < 0.05); nd = not detected.

## Data Availability

The data are contained within this article.
